# Common Muscle Metabolic Signatures Highlight Arginine and Lysine Metabolism as Potential Therapeutic Targets to Combat Unhealthy Aging

**DOI:** 10.3390/ijms22157958

**Published:** 2021-07-26

**Authors:** Janina Tokarz, Gabriele Möller, Anna Artati, Simone Huber, Anja Zeigerer, Bert Blaauw, Jerzy Adamski, Kenneth Allen Dyar

**Affiliations:** 1 Institute for Diabetes and Cancer, Helmholtz Zentrum München, German Research Center for Environmental Health, 85764 Neuherberg, Germany; janina.tokarz@helmholtz-muenchen.de (J.T.); gabriele.moeller@helmholtz-muenchen.de (G.M.); anja.zeigerer@helmholtz-muenchen.de (A.Z.); 2 German Center for Diabetes Research (DZD), 85764 Neuherberg, Germany; 3 Metabolomics and Proteomics Core, Helmholtz Zentrum München, German Research Center for Environmental Health, 85764 Neuherberg, Germany; anna.artati@helmholtz-muenchen.de (A.A.); simone.huber@helmholtz-muenchen.de (S.H.); 4 Joint Heidelberg-IDC Translational Diabetes Program, Inner Medicine 1, Heidelberg University Hospital, 69120 Heidelberg, Germany; 5 Department of Biomedical Sciences, University of Padova, 35129 Padova, Italy; bert.blaauw@unipd.it; 6 Venetian Institute of Molecular Medicine, 35129 Padova, Italy; 7 Institute of Experimental Genetics, Helmholtz Zentrum München, German Research Center for Environmental Health, 85764 Neuherberg, Germany; adamski@helmholtz-muenchen.de; 8 Institute of Biochemistry, Faculty of Medicine, University of Ljubljana, Vrazov Trg 2, 1000 Ljubljana, Slovenia; 9 Department of Biochemistry, Yong Loo Lin School of Medicine, National University of Singapore, Singapore 117596, Singapore

**Keywords:** skeletal muscle, metabolomics, diet, exercise, lifestyle, aging, metabolic signatures

## Abstract

Biological aging research is expected to reveal modifiable molecular mechanisms that can be harnessed to slow or possibly reverse unhealthy trajectories. However, there is first an urgent need to define consensus molecular markers of healthy and unhealthy aging. Established aging hallmarks are all linked to metabolism, and a ‘rewired’ metabolic circuitry has been shown to accelerate or delay biological aging. To identify metabolic signatures distinguishing healthy from unhealthy aging trajectories, we performed nontargeted metabolomics on skeletal muscles from 2-month-old and 21-month-old mice, and after dietary and lifestyle interventions known to impact biological aging. We hypothesized that common metabolic signatures would highlight specific pathways and processes promoting healthy aging, while revealing the molecular underpinnings of unhealthy aging. Here, we report 50 metabolites that commonly distinguished aging trajectories in all cohorts, including 18 commonly reduced under unhealthy aging and 32 increased. We stratified these metabolites according to known relationships with various aging hallmarks and found the greatest associations with oxidative stress and nutrient sensing. Collectively, our data suggest interventions aimed at maintaining skeletal muscle arginine and lysine may be useful therapeutic strategies to minimize biological aging and maintain skeletal muscle health, function, and regenerative capacity in old age.

## 1. Introduction

Biological aging involves a gradual functional deterioration across multiple organ systems, eventually resulting in tissue dysfunction [1]. Accordingly, age is the main risk factor for all major debilitating and life-threatening conditions of the developed world, including cancer, cardiovascular disease, neurodegeneration, type 2 diabetes, and musculoskeletal disorders like sarcopenia and osteoporosis. Globally, ‘people over the age of 65’ is the fastest growing segment of the population [2], yet most over 65 live with some kind of chronic illness, disability, or frailty that negatively impacts their quality of life (health span), restricts their ability to be economically or socially active, and dramatically increases their healthcare costs [3]. In order to preserve and improve the health and vitality of our rapidly aging global population, there is a recognized need to identify, understand, and promote various mechanisms for healthy biological aging (e.g., nutrition, physical activity, senotherapeutics/geroprotectors) and to detect and minimize unhealthy aging trajectories.

How biological aging unfolds is a uniquely personal process, and depends on a lifetime of complex sequential and cumulative interactions between genes and environment. Nevertheless, researchers have identified several common cellular and molecular traits associated with biological aging. These hallmarks [1] include primary causes of tissue damage (mitochondrial dysfunction, oxidative stress, DNA damage, epigenetic modifications, and telomere attrition), and secondary responses and repercussions (deregulated nutrient sensing, cellular senescence, chronic inflammation, and stem cell dysfunction and depletion).

Importantly, all established hallmarks are linked to metabolism [4], and evidence suggests that a ‘rewired’ metabolic circuitry [5] can accelerate (hypercaloric diet, inactivity) or delay (caloric restriction, exercise) biological aging. For example, a high fat diet (HFD) causes obesity in mice and is associated with all classic hallmarks of biological aging, including telomere attrition, epigenetic alterations, mitochondrial dysfunction, senescence, deregulated nutrient sensing, altered intracellular communication, genomic instability, and loss of proteostasis [6]. Likewise, aging hallmarks can be reduced in several animal models under caloric restriction and various other dietary interventions [7]. In mice, longevity-promoting effects of caloric restriction varies according to genetic background and is thought to stem from the prevention of excessive body mass and adiposity, and overall reductions in oxidative stress [8].

Musculoskeletal dysfunctions are among the most common comorbidities associated with aging [9]. A progressive decline in muscle mass and force (sarcopenia) and reduced muscle stem cell (satellite cell) proliferation makes aged muscles more susceptible to damage and slower to regenerate and recover [10,11]. On the other hand, diet and exercise (lifestyle) are established preventative measures to maintain a healthy aging trajectory and quality of life. This is in part by maintaining mitochondrial function, including redox homeostasis [12] and nutrient sensing, and by combatting the age-associated loss of muscle mass and strength [13,14,15].

Metabolomics has proven to be a powerful tool to establish age-related metabolic signatures in humans [16] and mice [17]. This is because the presence and relative abundance of certain metabolites can serve as diagnostic indicators (biomarkers). Designation of several diagnostic metabolites has already allowed clinicians to confidently define health status, predict future disease risk, track disease progression, and chart the progress of various therapeutic interventions [18]. While metabolic signatures in blood and urine can non-invasively differentiate diseases, and may reflect various aspects of tissue-specific dysfunction and pathology, they are no substitute for directly measuring the diseased tissue. Indeed, recent human studies comparing metabolites in blood and muscle biopsies from the same subjects explicitly discouraged the use of blood metabolites as direct surrogates for muscle metabolism [19]. Accordingly, many current predictive risk biomarkers may only reflect indirect, distant metabolic consequences of the diseases they describe, rather than direct causes. While this does not necessarily impact their diagnostic value, this can severely limit their utility in disease prevention and maintenance, and for uncovering pathophysiological mechanisms.

To identify metabolic signatures in skeletal muscle that distinguish healthy from unhealthy biological aging trajectories, we performed nontargeted metabolomics in skeletal muscles from different experimental cohorts of C57BL/6 mice. We hypothesized that common metabolic signatures would highlight specific pathways and processes that promote healthy aging, while simultaneously revealing the molecular underpinnings of unhealthy aging. We therefore investigated the skeletal muscle metabolome at different ages and under different interventions known to impact biological aging (i.e., diet and exercise) using ultra high performance liquid chromatography/mass spectrometry (UPLC-MS/MS). Here, we present 50 metabolites that commonly distinguish healthy from unhealthy trajectories. We have compiled these into an ‘Advanced Age Muscle-Enriched Metabolite Set’ (AAMEMS). This metabolite set can be used for enrichment analysis to interrogate and qualify skeletal muscle metabolomics datasets according to healthy and unhealthy trajectories. Collectively, our data suggest interventions aimed at maintaining skeletal muscle arginine and lysine may be useful therapeutic strategies to minimize biological aging and maintain skeletal muscle health, function, and regenerative capacity in old age.

## 2. Results

### 2.1. Changes in the Skeletal Muscle Metabolome Associated with Age, Diet and Lifestyle

**Figure 1:**
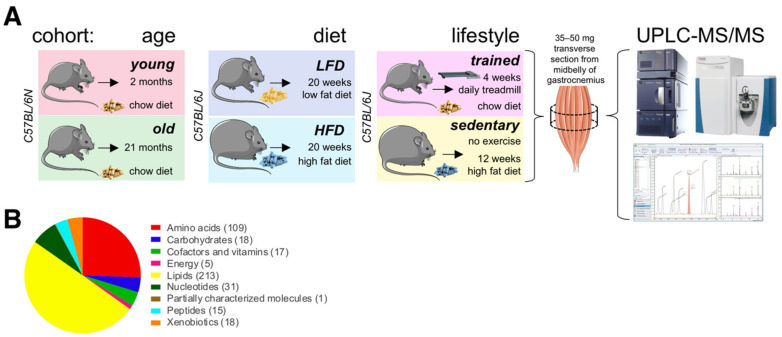
The skeletal muscle metabolome in relation to age, diet, and lifestyle. (**A**) Scheme of experimental design. Figure elements modified from SMART (Servier Medical Art), licensed under a Creative Common Attribution 3.0 Generic License. http://smart.servier.com/ accessed on 9 June 2021. (**B**) Classification of 427 detected metabolites. The number of metabolites per class is given in parentheses.

We profiled and compared relative abundance of metabolites in *gastrocnemius* muscles from different experimental cohorts of wildtype C57BL/6 mice (Figure 1A). To investigate the impact of age, we collected muscles from 2-month and 21-month-old male mice fed the same chow diet (14 kcal% fat, 59 kcal% carbohydrate, 27 kcal% protein; 3.34 kcal/g). We chose two months of age to replicate a prior study [20] and to capture a metabolic snapshot at the beginning of adulthood, when rapid postnatal muscle growth begins to stabilize [21,22]. We chose 21-months as the beginning of old age, when loss of muscle mass and function begins due to age-associated sarcopenia [20,23]. We collected tissues under basal metabolic conditions at ZT6 (zeitgeber time 6, noon) after a 4 h fast and during the normal physiological fasting + rest phase for nocturnal mice [24].

To investigate how diet impacts the muscle metabolome, we also profiled muscles from 7-month-old male mice fed either a low fat diet (LFD; 10 kcal% fat, 70 kcal% carbohydrate, 20 kcal% protein; 3.82 kcal/g) or a high fat diet (60 kcal% fat, 20 kcal% carbohydrate, 20 kcal% protein; 5.21 kcal/g) for 20 weeks. Again, we collected tissues under basal metabolic conditions at ZT6 after a 4 h fast. To investigate possible interactions between exercise, nutrition, and age-associated metabolic signatures (lifestyle), we also profiled muscle metabolites from male endurance trained mice and sedentary littermates fed different diets. For this cohort, we chose two well-characterized treatment regimens to maximize metabolic differences between groups at the time of tissue collection. Endurance trained mice ate a high fiber chow diet rich in complex carbohydrates (9 kcal% fat, 68 kcal% carbohydrate, 23 kcal% protein; 3.15 kcal/g) and ran on a motorized treadmill for 1 h at 68% of their maximal workload seven days a week for four weeks. This kind of moderate intensity exercise has been shown to produce beneficial cardiovascular and skeletal muscle adaptations in mice [25] and is similar to recommendations for optimal fitness in humans [26,27]. To maintain normal circadian rhythms, training was always performed under dim red light starting at around ZT12 (zeitgeber time 12; 6 pm when lights are turned off in the animal facility) to coincide with the start of the circadian activity phase. Sedentary littermates did not exercise, and ate a highly processed high fat diet (60 kcal% fat, 20 kcal% carbohydrate, 20 kcal% protein; 6.0 kcal/g) for 12 weeks. While interventions began at different stages (starting at 4 months of age for sedentary mice on high fat diet, and at 6 months for endurance trained mice on chow diet), littermates were all 7-month-old at the time of tissue collection. We collected all tissues under basal metabolic conditions at ZT11, prior to awakening at the end of the normal physiological fasting + rest phase. To directly compare relative metabolite levels between these three groups (‘age’, ‘diet’ and ‘lifestyle’), we randomized all tissue samples, and then processed, extracted, and measured them all together in a single analytical set. We detected 427 metabolites, including a wide range of amino acids, carbohydrates, lipids, nucleotides, peptides, energy metabolites linked to the TCA cycle, cofactors, vitamins, and xenobiotics (Figure 1B).

### 2.2. Age-Associated Changes in the Skeletal Muscle Metabolome

**Figure 2:**
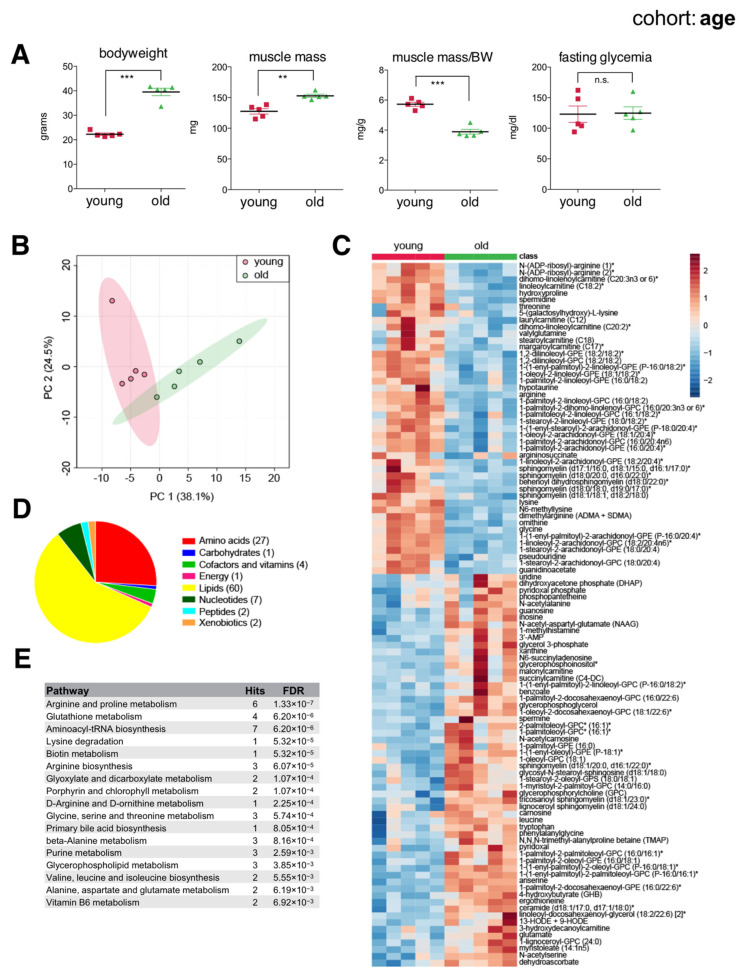
The skeletal muscle metabolome reflects changes associated with age. (**A**) Bodyweight, muscle weight (*gastrocnemius*), muscle weight relative to bodyweight (BW), and glycemia after a 4 h fast of young (2-month) and old (21-month) male C57BL/6N mice (mean ± SEM; *n* = 5; *** *p* < 0.0001, ** *p* = 0.0011, n.s. not significant, Student’s *t*-test). (**B**) Principal component analysis of the entire metabolomics data set. (**C**) Heatmap of metabolites selected by random forest shown for individual samples. Metabolites labeled with an asterisk have not been confirmed with a standard, but we are confident of their identity. (**D**) Classification of random forest selected metabolites to their metabolite class. The number of metabolites per class is given in parentheses. (**E**) Quantitative enrichment analysis showing the top enriched pathways with FDR < 0.01 based on the metabolites selected by random forest.

Older mice had greater bodyweight and muscle mass compared to younger mice. However, 21-month-old mice already showed a significantly reduced ratio of muscle mass to bodyweight (Figure 2A), reflecting increased adiposity and suggesting overall reduced muscle function. Principal component analysis (PCA) of metabolites showed separation according to age (Figure 2B). To classify metabolites according to age, we applied random forest and found 104 significant metabolites distinguishing young from old muscles, more than twice the number of metabolites previously detected in similar studies [20,28]. These included 46 metabolites of reduced concentration and 58 of increased concentration in aged muscles, consisting mainly of membrane lipids, acylcarnitines, ceramides, amino acids, and polyamines (Figure 2C–E). Of note, aged muscles showed reduced levels of long and medium chain acylcarnitines like linoleoylcarnitine (C18:2), stearoylcarnitine (C18), and laurylcarnitine (C12), yet an increased abundance of short chain acylcarnitines like succinylcarnitine (C4-DC) and malonylcarnitine (C3-DC). Arginine and lysine metabolites were also reduced in aged muscles, while leucine, ceramides, nucleosides (guanosine, inosine, uridine), purine catabolites (xanthine), and various free radical scavengers (carnosine, anserine, ergothioneine) were all increased. Accumulation of antioxidants in aged muscles suggested a response to increased oxidative stress. Indeed, we also noted increased hydroxyoctadecadienoic acids (13-HODE + 9-HODE), stable oxidation products of linoleic acid, and markers of oxidative stress that are known to accumulate with age [29]. Overall, our data highlighted differences in basal ‘metabolic wiring’ distinguishing young and aged skeletal muscles largely consistent with previous studies, as well as additional metabolic players that were previously unknown.

### 2.3. Diet-Associated Changes in the Skeletal Muscle Metabolome

**Figure 3:**
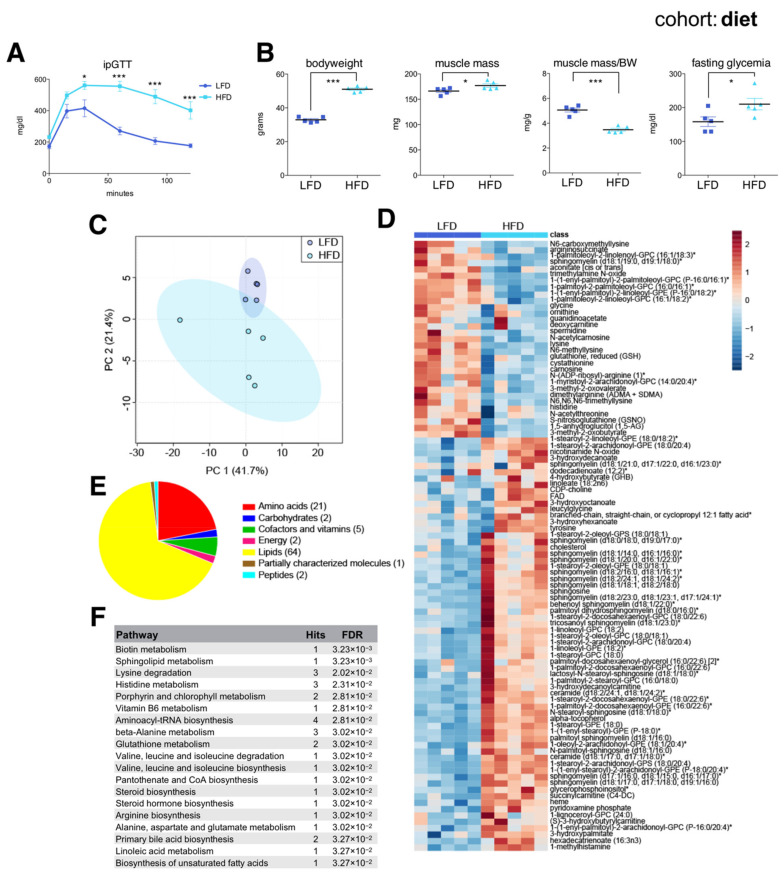
The skeletal muscle metabolome reflects changes associated with diet. (**A**) Intraperitoneal glucose tolerance test (ipGTT) performed one week prior to sac in the afternoon, after a 6 h fast in seven-month-old C57BL/6J male mice on a low fat diet (LFD) or a high fat diet (HFD) (mean ± SEM; *n* = 6; * *p* < 0.05, *** *p* < 0.001, 2-way ANOVA with Bonferroni correction; diet effect F = 26.60, *p* < 0.0004; time effect F= 39.06, *p* = 0.0001; diet × time interaction F = 7.82, *p* < 0.0001). (**B**) Bodyweight, muscle weight (*gastrocnemius*), muscle weight relative to bodyweight (BW), and glycemia after a 4 h fast (mean ± SEM; *n* = 5; *** *p* < 0.0001, * *p* < 0.05, Student’s *t*-test). (**C**) Principal component analysis of the entire metabolomics data set. (**D**) Heatmap of metabolites selected by random forest shown for individual samples. Metabolites labeled with an asterisk have not been confirmed with a standard, but we are confident of their identity. (**E**) Classification of random forest selected metabolites according to metabolite class. The number of metabolites per class is shown in parentheses. (**F**) Quantitative enrichment analysis showing the top enriched pathways with FDR < 0.05 based on the metabolites selected by random forest.

As expected, HFD-fed mice were severely glucose intolerant (Figure 3A) and had increased bodyweight, muscle mass, and fasting glycemia compared to LFD-fed mice (Figure 3B). HFD-fed mice had a decreased muscle mass to bodyweight ratio, reflecting their massively increased adiposity. Muscle metabolomics distinguished each group according to diet (Figure 3C), with random forest identifying 96 significant diet-associated metabolites: 31 were reduced and 65 increased under HFD (Figure 3D). Similar to metabolites associated with old age, these were mainly membrane lipids, acylcarnitines, ceramides, and amino acids (Figure 3E,F). Similar to aging, HFD was associated with reduced muscle levels of glycine and several metabolites related to arginine and lysine metabolism, including spermidine and the creatine precursor guanidinoacetate, and with an increased abundance of various ceramides and short chain acylcarnitines (succinylcarnitine). These results were largely consistent with previous data [30], and verified that skeletal muscles from younger high fat diet-induced obese mice share many common metabolic features associated with biological aging.

### 2.4. Lifestyle-Associated Changes in the Skeletal Muscle Metabolome

**Figure 4:**
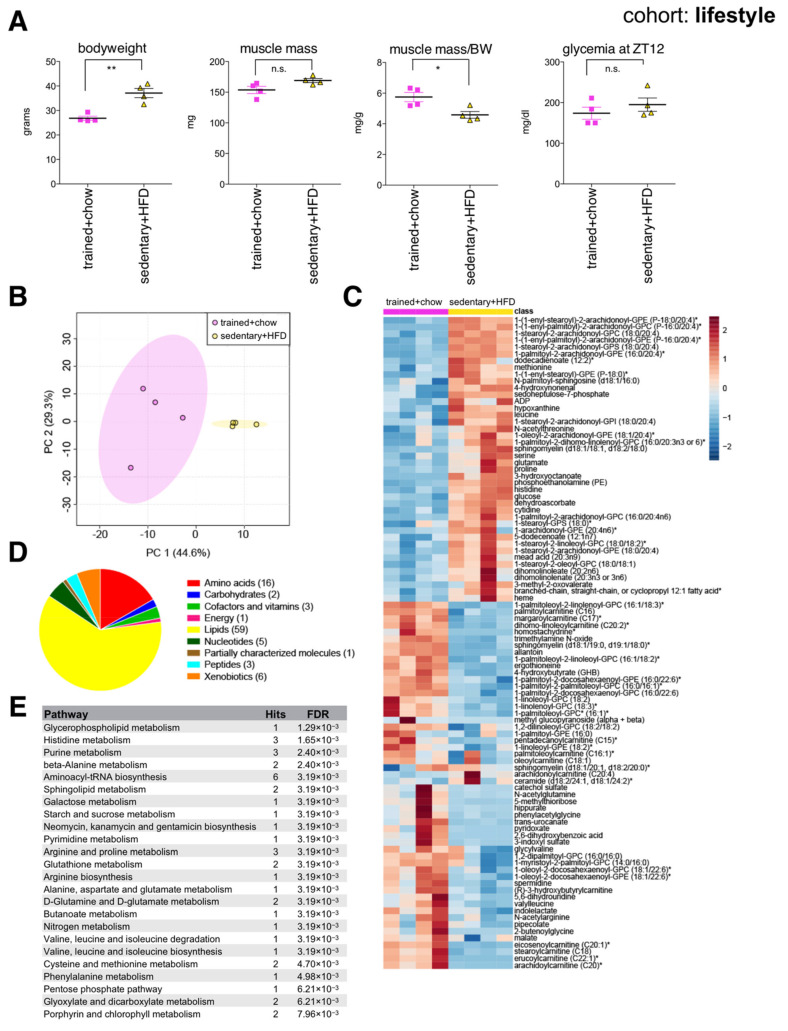
The skeletal muscle metabolome reflects changes associated with lifestyle. (**A**) Bodyweight, muscle weight (*gastrocnemius*), muscle weight relative to bodyweight (BW), and glycemia at zeitgeber time 12 (ZT12, 6 pm) of male C57BL/6J mice after five weeks of daily endurance training (1 h on a motorized treadmill) and eating a chow diet, or sedentary littermates after three months eating a high fat diet (HFD) (mean ± SEM; *n* = 4; ** *p* < 0.01, * *p* < 0.05, n.s. not significant, Student’s *t*-test). (**B**) Principal component analysis of the entire data set. (**C**) Heatmap of metabolites selected by random forest shown for individual samples. Metabolites labeled with an asterisk have not been confirmed with a standard, but we are confident of their identity. (**D**) Classification of random forest selected metabolites to their metabolite class. The number of metabolites per class is given in parentheses. (**E**) Quantitative enrichment analysis showing the top enriched pathways with FDR < 0.01 based on the metabolites selected by random forest.

Compared to endurance trained mice, sedentary littermates on HFD had greater body mass, similar muscle mass, yet significantly reduced muscle mass to bodyweight ratio (Figure 4A). Interestingly, 7-month-old endurance trained mice showed a similar ratio of muscle mass to bodyweight to 2-month-old mice (Figure 2A), reflecting their reduced adiposity, and maintenance of overall skeletal muscle health. Muscle metabolites distinguished each group according to lifestyle (Figure 4B), although the endurance trained group showed greater variance between individuals. Random forest identified 96 significant lifestyle-associated metabolites, with 54 reduced and 42 increased in the sedentary HFD-fed group (Figure 4C). Again, a variety of membrane lipids distinguished each group (Figure 4D,E). Muscles from sedentary HFD-fed mice again showed reduced spermidine, similar to aged and HFD muscles. Muscles from endurance trained mice on chow diet showed increased abundance of various long chain acylcarnitines, generally reduced amino acids, and lower glucose compared to the sedentary HFD-fed group. This reflects expected metabolic adaptations associated with endurance training, and suggests muscles from endurance trained mice use more lipids as energy substrates. Interestingly, similar metabolic signatures in human plasma have been reported in marathon runners in the days after a race [31]. 

### 2.5. Common Skeletal Muscle Metabolite Signatures Distinguish ‘Desirable’ from ‘Undesirable’ Aging Trajectories

**Figure 5:**
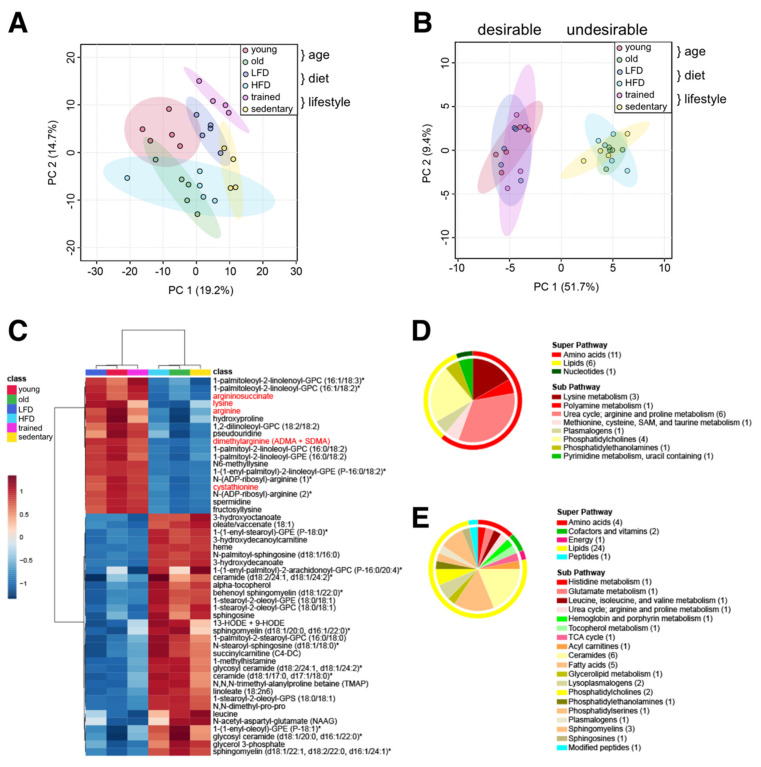
Common muscle metabolite signature distinguishes ‘desirable’ from ‘undesirable’ metabolic states. (**A**) Principal component analysis of all 427 metabolites in all three data sets. (**B**) Principal component analysis of 50 common metabolites identified by meta-analysis. (**C**) Heatmap of 50 common metabolites identified by meta-analysis. Group averages are presented. Metabolites labeled with an asterisk have not been confirmed with a standard, but we are confident of their identity. Metabolites localized in mitochondria are labelled red. (**D**,**E**) Classification of metabolites decreased (**D**) or increased in the ‘undesirable’ metabolic state (**E**) according to the super pathway (outer circle) and the sub pathway (inner circle). The number of metabolites per class is given in parentheses.

Treatments aimed at suppressing specific risk metabolites can prevent diseases and improve clinical outcomes, even when patients are otherwise asymptomatic [32]. To identify common metabolites distinguishing healthy from unhealthy aging trajectories, we combined our three datasets (‘age’, ‘diet’, and ‘lifestyle’) and investigated them collectively. We hypothesized that common metabolic signatures would highlight specific pathways and processes that promote healthy aging, while revealing the molecular underpinnings of unhealthy aging. Principal component analysis (PCA) performed on all 427 detected metabolites showed overlap between muscles from old mice and both HFD-fed cohorts (Figure 5A). On the other hand, muscles from endurance trained mice (treadmill), young mice, and LFD-fed mice showed more discrete clusters, suggesting they shared fewer common metabolic signatures.

Performing a statistical meta-analysis of all 3 groups, we identified 50 metabolites that commonly distinguished ‘desirable’ (young, LFD, and trained) from ‘undesirable’ metabolic states (old, HFD, and sedentary) (Figure 5B,C). These include 18 metabolites commonly reduced and 32 commonly increased in ‘undesirable’ states associated with advanced age. Interestingly, glycerophosphocholine (GPC) and glycerophosphoethanolamine (GPE) containing residues of palmitate (saturated), palmitoleate (omega-7 monounsaturated), or linoleate (omega-6 polyunsaturated) at the C-1 position, and linoleate or linolenate (omega-3 polyunsaturated) at the C-2 position were commonly reduced under ‘undesirable’ metabolic states. On the other hand, GPC and GPE containing stearate (saturated) and oleate (monounsaturated) residues were commonly increased in ‘undesirable’ conditions. Intriguingly, a variety of arginine-related metabolites (arginine, argininosuccinate, dimethylarginine, N-ADP-ribosyl-arginine, spermidine), lysine metabolites (lysine, N6-methyllysine, fructosyllysine), cystathionine, hydroxyproline, and pseudouridine were also commonly reduced in ‘undesirable’ conditions (Figure 5D). Commonly increased metabolites in ‘undesirable’ metabolic states reflected an increased oxidative environment, including stable markers of oxidative stress (13-HODE + 9-HODE), oxidants (heme), and antioxidants (alpha-tocopherol/vitamin E), in addition to glycerol 3-phosphate, leucine, short and medium chain acylcarnitines (succinylcarnitine, 3-hydroxydecanoylcarnitine), medium chain hydroxy fatty acids (3-hydroxyoctanoate, 3-hydroxydecanoate), oleate, linoleate, ceramides, sphingolipids, and methylated amino acids and peptides (1-methylhistamine, N,N-dimethyl-pro-pro, N,N,N-trimethyl-alanylproline betaine/TMAP) (Figure 5E). Interestingly, the neuropeptide N-acetylaspartylglutamate (NAAG) was also increased in all ‘undesirable’ metabolic states, suggesting shared functional alterations in neurotransmission or changes in the neuromuscular junction [33]. 

**Figure 6:**
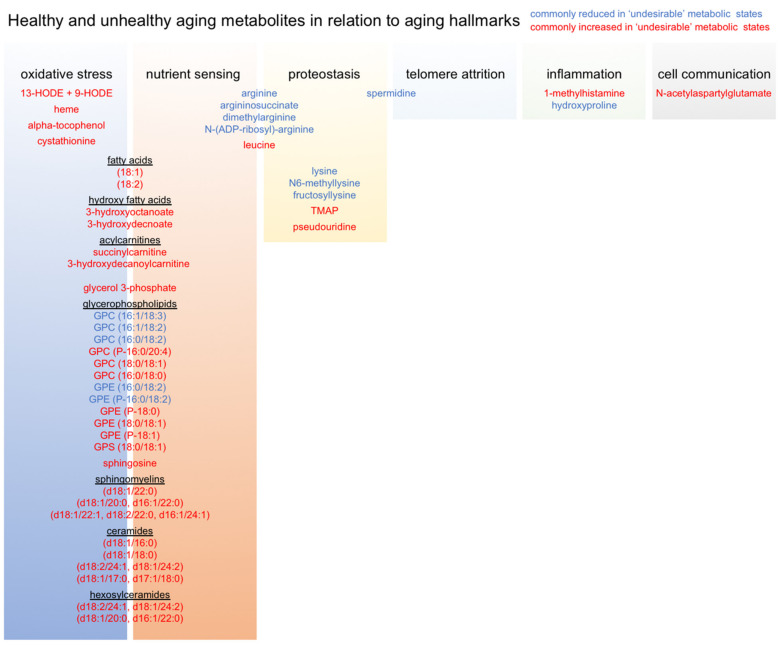
Relationships between metabolites associated with healthy and unhealthy aging trajectories and aging hallmarks.

Finally, we compiled and manually curated a list of all common metabolites associated with our healthy and unhealthy aging trajectories in order to generate an ‘Advanced Age Muscle-Enriched Metabolite Set’ (AAMEMS). This list (Supplementary Table S10) can be used for metabolite enrichment analysis of new or previously published metabolomics datasets in order to qualify metabolic signatures in terms of healthy and unhealthy aging trajectories. After stratifying these metabolites according to known relationships with various aging hallmarks, we noted that our metabolic signature of unhealthy aging was mostly associated with oxidative stress and nutrient sensing (Figure 6). This suggests that these processes are especially important metabolic drivers of biological aging, and highlights them as particularly useful targets for therapeutic interventions, whether by lifestyle, nutrition, or pharmacologically. Our data also underscores the importance of lifelong mindfulness of diet and physical activity for prevention of unhealthy aging trajectories [34,35,36].

## 3. Discussion

Research into the origins of biological aging is expected to reveal modifiable molecular mechanisms that can be harnessed to slow or possibly even reverse unhealthy aging trajectories. However, before this future can be realized there is an urgent need to define consensus molecular biomarkers of healthy and unhealthy biological aging. Complicating matters, these markers are likely to be context-specific (i.e., depending on sex or genetic background) and may differ according to cell-type, subcellular localization, or the presence and severity of various age-related comorbidities and/or therapies.

Here, we produced and explored three different in vivo experimental datasets to uncover a common skeletal muscle metabolic signature distinguishing healthy from unhealthy aging in mice. Our common signature consists of 50 metabolites, with 18 metabolites commonly reduced in ‘undesirable’ metabolic states and 32 increased. We have compiled these metabolites into an ‘Advanced Age Muscle-Enriched Metabolite Set’ (AAMEMS) that can be used to qualify previous and future muscle metabolomics datasets in terms of healthy and unhealthy aging trajectories.

We detected these particular metabolites in muscles from male mice under fasting conditions with a particular nontargeted metabolomics method. Accordingly, our results are likely just the proverbial tip of the iceberg. Future studies using additional detection methods (whether broader or more targeted) or broader experimental designs (i.e., profiling female mice, or including temporal profiling to capture more dynamic metabolic signatures) are warranted to add additional information and physiological context. Validation studies, including quantitative assays performed in diverse human cohorts, and exploring whether similar metabolite alterations can be detected in blood, are also required to verify the translational and clinical relevance of this particular metabolic signature.

Indeed, while we identified many new metabolites associated with unhealthy aging that will require further examination, our findings are largely consistent with previously published rodent and human studies focused on skeletal muscle aging in general. For example, Houtkooper and colleagues [28] similarly detected increased linoleate, leucine, and glycerol 3-phosphate with reduced hydroxyproline in quadriceps from 103 weeks old versus 24 weeks old C57BL/6J mice (sex unreported) after an overnight fast. More recently, Uchitomi and colleagues [20] reported increased 1-methylhistamine and various neurotransmitters suggestive of neuromuscular junction degeneration in *gastrocnemius* muscles from 28-month-old versus 2-month-old C57BL/6J male mice fed ad libitum (neither time of day nor nutritional state at the time of tissue collection was reported). In agreement with our data, they also reported reduced hydroxyproline, N6-methyllysine, and spermidine in aged muscles. Garvey and colleagues [37] also reported increased sphingosine, glycerol 3-phosphate, leucine, linoleate, oleate, and cis-vaccenate, with reduced hydroxyproline in *gastrocnemius* and *soleus* muscles from 32-month-old versus 15-month-old male rats fasted for 16 h. Interestingly, Garvey et al. also reported that plasma heme levels showed the largest age-related increase, which is in agreement with our muscle data. A recent human nontargeted metabolomics study compared *vastus lateralis* muscle biopsies collected from young (25 ± 4 y), middle aged (50 ± 4 y), and older (70 ± 3 y) males and females (50:50) of similar lean mass after an overnight fast [38]. In general agreement with our results, their metabolite network of human muscle aging centered around histamine, phospholipids, polyamines, phosphocreatine (also linked to arginine metabolism), and androgens (not detected by our method). Another human study by Fazelzadeh and colleagues [19] found similarly increased 9-HODE and 13-HODE and reduced hydroxyproline in biopsies from *vastus lateralis* collected in the morning after an overnight fast from healthy aged (71.7 ± 5.2 y; BMI 25.5 ± 3.0) men (*n* = 47) and women (*n* = 19) compared to younger men (21.7 ± 2.5 y; BMI 22.6 ± 2.7; *n* = 30). However, in contrast to our data, the authors noted reduced leucine and short chain acylcarnitines (malonylcarnitine, C3-DC), and increased arginine and lysine in healthy aged muscles. Further comparing healthy aged subjects to frail aged (77.5 ± 8.0 y; BMI 27.5 ± 3.7) males (*n* = 25) and females (*n* = 18), the authors also noted reduced malonylcarnitine, yet increased spermidine in the frail subjects. These inconsistencies with our data are somewhat puzzling, and may reflect sex-dependent differences among heterogeneous aged populations compared to young males, or differences in collection times (during the awake phase in humans) and general changes in leucine, arginine, and lysine 24 h dynamics. Spermidine is generally thought to be reduced with aging, but the authors speculated that higher spermidine concentrations in frail subjects may reflect differences in underlying neuromuscular disorders or oxidative stress. Finally, similar to our data uncovering metabolic signatures of unhealthy aging trajectories, linoleate and alpha-tocopherol were both reported to be elevated in human *vastus lateralis* muscles from overweight twins after a 12 h fast [39].

Skeletal muscle membrane lipids have also previously been tied to biological age [20,28]. This may be expected, as sarcolemmal and mitochondrial membrane lipid composition are known to be altered by diet [40,41], exercise [42], and aging [43]. Specific changes in omega-3 and omega-6 polyunsaturated fatty acids like the ones we found are functionally linked to decreased lipid transport and oxidation, reductions in ADP sensitivity, increased susceptibility to oxidative damage, and reduced insulin sensitivity for skeletal muscle anabolism (for review see [44]). It is well known that polyunsaturated fatty acids in membrane phospholipids are particularly susceptible to oxidative damage by free radicals. Therefore, the elevated linoleate (C18:2) we identified in all ‘undesirable’ experimental groups likely reflects increased oxidative stress under these conditions. Similarly, linoleate was found to be increased in quadriceps from obese/diabetic versus lean/diabetic mice [45], in rat *plantaris* muscles after an acute bout of exercise [46], and in a time-dependent manner in *tibialis anterior* muscles from circadian clock disrupted mice [24].

We also uncovered several new signatures of unhealthy biological aging that require further clarification. In all three ‘undesirable’ metabolic states, we found increased leucine with reduced arginine and lysine metabolites. The origins and relative functional impacts of these changes remain to be elucidated, but aging may alter processes involved in their uptake, transport, and/or interorgan flux. For example, aging is known to reduce the sensitivity of skeletal muscle protein synthesis to dietary amino acids, and the elderly require increased dietary protein and/or essential amino acids to stimulate the same synthesis rates observed in younger individuals [47]. It is also well known that among amino acids, leucine and arginine are the most potent activators of mammalian target of rapamycin complex 1 (mTORC1) [48], a metabolic rheostat [49] and central signaling hub that controls anabolic and catabolic processes according to nutrient availability. Skeletal muscles from aged mice show increased phosphorylation of the ribosomal protein S6, indicative of increased mTORC1 activation [50]. On the other hand, inhibition of mTORC1 with rapamycin increases lifespans [51] in part by reducing protein and lipid biosynthetic pathways and by activating protein quality control mechanisms, like autophagy. Mice treated long-term with rapamycin are largely spared sarcopenia and maintain skeletal muscle mass, function, and neuromuscular junction integrity into old age [33]. Although diets supplemented with leucine or arginine promote muscle growth via increased mTORC1 activity [52], they do so via distinct mechanisms [53,54,55]. Intriguingly, lysine supplementation in growing piglets was recently shown to enhance satellite cell proliferation and skeletal muscle protein synthesis also in an mTORC1-dependent manner [52]. To verify the prognostic value of leucine, arginine, and lysine metabolites as bona fide biomarkers of healthy aging, and to establish healthy and unhealthy diagnostic ranges and cut-offs, future targeted studies measuring absolute quantification in muscles and blood from large and diverse patient cohorts are required.

However, the therapeutic potential of some changes we identified is already suggested by previous studies. Leucine supplementation in sarcopenic patients seems to provide only modest effects in the maintenance of muscle mass [56,57,58]. However, maintenance of muscle mass, strength, and function are all enhanced in elderly subjects when leucine is given in combination with arginine [59,60] and other essential amino acids like lysine [61]. Furthermore, lysine supplementation has been shown to suppress autophagic myofibrillar protein degradation in skeletal muscles, both in rats [62] and in senescence-accelerated sarcopenic mice [63].

Argininosuccinate, lysine, arginine, dimethylarginine (ADMA + SDMA) and cystathionine are all present in mitochondria [64], so their reduced abundance in ‘undesirable’ metabolic states may simply reflect lower mitochondrial content in aged muscles [65]. We previously found that muscle levels of arginine and lysine oscillate ~2-fold over 24 h in a circadian clock-dependent fashion [24]. Since high fat diet and aging are both associated with dampened circadian rhythms [30,66,67], reduced arginine and lysine in ‘undesirable’ metabolic states may likewise reflect a time-dependent decrease linked to altered circadian clock function.

Overall, our data suggests that strategies aimed at maintaining normal arginine and/or lysine levels in aged skeletal muscles may be particularly useful to combat age-associated sarcopenia and maintain the regenerative capacity of skeletal muscle. The full extent of this potential remains to be verified experimentally, as effects are likely to be multifactorial, likely extend beyond skeletal muscle, and may be problematic depending on the presence or severity of various comorbidities. However, there is already indication that this kind of approach is worthy of further exploration. For example, arginine is known to have a wide range of physiological functions throughout the body, including stimulating growth hormone secretion [68], and acting as a precursor for nitric oxide, creatine, agmatine, and spermidine biosynthesis [69]. Spermidine, which we found reduced in skeletal muscles under all ‘undesirable’ metabolic states, is also known to decline with age. Spermidine supplementation has been shown to increase lifespan in worms and flies [70], and reduce age-related pathologies in brain, liver, heart, and kidney of aged mice [71], including telomere attrition. Increasing systemic arginine may also improve many important physiologic processes that decline with aging, including tissue perfusion, immune system function, protein synthesis, and wound healing [72]. 

In conclusion, our data highlight several common modifiable skeletal muscle metabolic alterations linked to aging and underscore the fundamental roles diet and physical activity play in prevention and correction of unhealthy aging trajectories. Expanding upon previously known hallmarks and biochemical signatures of aging, we identified arginine and lysine metabolites, and their related metabolic processes, as common metabolic hubs and potential new players driving biological aging.

## 4. Materials and Methods

### 4.1. Mice

Male C57BL/6 mice were housed in a controlled environment (12 h light/12 h dark, ~23 °C), with ad libitum access to food and water. We randomly assigned littermates to each experimental group. Blood glucose was measured at the time of tissue collection using a glucometer (AccuCheck Aviva, Roche Diagnostics, Risch-Rotkreuz, Switzerland). Details of experimental groups are listed in Table 1.

### 4.2. Endurance Training

For endurance training, mice trotted 25 cm/s and 0% incline on a motorized treadmill (Harvard Apparatus PANLAB, LE 8710 M, Holliston, MA, USA) 1 h daily, 7 days/week for 4 weeks. This intensity was 68% of their maximal workload attained during an exercise performance test. After humanely encouraging mice to run with a soft brush on their tail during the first few acclimation sessions, mice ran without much need of further encouragement. 

### 4.3. Intraperitoneal Glucose Tolerance Test

A glucose tolerance test was performed in LFD and HFD mice one week prior to tissue collection. Mice were fasted for 6 h before analysis during their normal physiological rest/fasting phase. Blood glucose was measured via the tail vein using a glucometer (AccuCheck Aviva, Roche Diagnostics, Risch-Rotkreuz, Switzerland) at 0, 15, 30, 60, 90, and 120 min after mice were injected i.p. with glucose (2 g/kg bodyweight).

### 4.4. Muscle Tissue Collection and Preparation for Metabolomics

*Gastrocnemius* muscles were collected immediately after cervical dislocation, snap frozen in liquid nitrogen, and stored at −80 °C until subsequent use. We randomized the samples and processed them all together as a single sample set split between three analytical batches. Transverse pieces (~35–50 mg) from the midbelly region were first homogenized with 1.4 mm ceramic beads in water (15 µL/mg) at 4 °C. To extract metabolites and to precipitate the protein, 500 µL methanol extraction solvent containing recovery standard compounds was added to each 100 µL of tissue homogenate. Supernatants were aliquoted and dried under nitrogen stream (TurboVap 96, Zymark, Hopkington, MA, USA) and stored at −80 °C until the UPLC-MS/MS measurements were performed.

### 4.5. Nontargeted Metabolomics

Two (i.e., early and late eluting compounds) aliquots were dedicated for analysis by UPLC-MS/MS in electrospray positive ionization and one for analysis by UPLC-MS/MS in negative ionization. Three types of quality control samples were included in each plate: samples generated from a pool of human ethylenediamine tetraacetic acid (EDTA) plasma, pooled sample generated from a small portion of each experimental sample served as technical replicate throughout the data set, and extracted water samples served as process blanks. Prior to UPLC-MS/MS analysis, the dried samples were reconstituted in acidic or basic LC-MS-compatible solvents, each of which contained eight or more isotopically labeled standard compounds at fixed concentrations to ensure injection and chromatographic consistency. The UPLC-MS/MS platform utilized a Waters (Milford, MA, USA) Acquity UPLC with Waters UPLC BEH C18-2.1 mm × 100 mm, 1.7 µm columns, a Thermo Scientific (Waltham, MA, USA) Q Exactive high resolution/accurate mass spectrometer interfaced with a heated electrospray ionization (HESI-II) source, and an Orbitrap mass analyzer operated at 35,000 mass resolution. One aliquot of the extracts was reconstituted in acidic positive ion conditions, chromatographically optimized for more hydrophilic compounds (for early eluting compounds). In this method, the extracts were gradient eluted from the C18 column using water and methanol containing 0.05% perfluoropentanoic acid (PFPA) and 0.1% formic acid (FA). Another aliquot that was also analyzed using acidic positive ion conditions, but was chromatographically optimized for more hydrophobic compounds (for later eluting compounds), was gradient eluted from the same C18 column using methanol, acetonitrile, and water; containing 0.05% PFPA and 0.01% FA and was operated at an overall higher organic content. The basic negative ion condition extracts were gradient eluted from a separate C18 column using water and methanol containing 6.5 mM ammonium bicarbonate at pH 8. The MS analysis alternated between MS and data dependent MS2 scans using dynamic exclusion and a scan range of 80–1000 m/z. Metabolites were identified by automated comparison of the ion features in the experimental samples to a reference library of chemical standard entries that included retention time, molecular weight (*m/z*), preferred adducts, and in-source fragments as well as associated MS spectra and curation by visual inspection for quality control using proprietary software developed by Metabolon Inc. Only fully annotated metabolites were included for further evaluation. Data were normalized according to raw area counts, and then each metabolite scaled by setting the median equal to 1. Missing data were imputed with the minimum. Biochemicals labelled with an asterisk (*) indicate compounds that have not been officially confirmed based on a standard, but we are confident in its identity.

### 4.6. Statistical Analysis and Quantitative Enrichment Analysis

Statistical data analysis including principal component analysis (PCA) of individual data sets was performed using the tool MetaboAnalyst 5.0 [73] using default parameters if not indicated otherwise. Data filtering was performed by the Interquartile Range (IQR) method. For selection of important features, Random Forest was applied (number of trees: 500, number of predictors: 20, randomness: use a constant). Metabolites with a mean decrease accuracy > 0 were selected and used to build a heatmap with MetaboAnalyst 5.0 and default heatmap parameters. This subset of metabolites was also used for quantitative enrichment analysis using the Enrichment Analysis module of MetaboAnalyst 5.0 [73] with the pathway-based KEGG metabolite set library (containing 84 metabolite sets based on human metabolic pathways). The metabolite classes of random forest selected metabolites were displayed as pie charts using GraphPad Prism 8.4.3 (GraphPad Software). Original data can be found in Supplementary Tables S1–S3, and metabolites selected by Random Forest in each data set can be found in Supplementary Tables S4–S6.

### 4.7. Statistical Meta-Analysis

To identify a common metabolite signature in all three data sets, statistical meta-analysis was performed using the Statistical Meta-Analysis module of MetaboAnalyst 5.0 [73]. In all data sets, the control samples were designated as ‘desirable’, while the remaining samples were designated as ‘undesirable’. Data were log_10_-transformed and auto-scaled (mean-centered and divided by the standard deviation). Differential expression analysis with a false discovery rate (FDR) of 0.1 and a fold change of 0.0 returned 158, 91, and 124 significant metabolites in the ‘age’, ‘diet’, and ‘lifestyle’ set, respectively. For meta-analysis, the combining *p*-values from multiple studies using the Stouffer’s method with a significance level of 0.01 was applied, which led to the identification of 50 significant metabolites (Supplementary Table S7). The transformed and scaled data for this subset of metabolites was used to build a PCA and a heatmap with MetaboAnalyst 5.0 using default parameters. The metabolite classes of metabolites based on their super and sub pathway classification by Metabolon Inc. were displayed as pie charts using GraphPad Prism 8.4.3 (GraphPad Software).

### 4.8. Generation of the AAMEMS-Library and Application in Quantitative Enrichment Analysis

To generate an ‘Advanced Age Muscle-Enriched Metabolite Set’ (AAMEMS) that can be used for enrichment analysis, we used the common set of 50 significantly changed metabolites identified by meta-analysis. The metabolites were manually matched to Human Metabolome Data Base (HMDB) names and identifiers (IDs), resulting in 38 metabolites with available HMDB IDs. These metabolites were divided into two sets; one set containing metabolites with increased levels of ‘undesirable’ skeletal muscle (22 metabolites, Supplementary Table S9) and one set containing metabolites with reduced levels of ‘undesirable’ skeletal muscle (16 metabolites, Supplementary Table S8). This AAMEMS-library was then uploaded as a user-defined metabolite set library for quantitative enrichment analysis in the Enrichment Analysis module of MetaboAnalyst 5.0 [73]. Five metabolites in the increased metabolite set were not matched by MetaboAnalyst. The final AAMEMS-library thus contains 17 metabolites with increased levels and 16 metabolites with decreased levels in skeletal muscles on an ‘undesirable’ aging trajectory (Supplementary Table S10). 

Our AAMEMS-library was then tested with the three data sets (Supplementary Table S11). A quantitative enrichment analysis (QEA) was performed using the Enrichment Analysis Module of MetaboAnalyst 5.0. Metabolite names were manually matched to HMDB IDs and the data was log-transformed and autoscaled. The final AAMEMS-library was uploaded as user-defined metabolite set. Similarly, we tested the AAMEMS-library on two previously published metabolomics data sets [20,28]. The metabolite names were manually matched to HMDB IDs, and the data was log-transformed and autoscaled and analyzed for enrichment of muscle-enriched metabolite sets using QEA and the Enrichment Analysis module of MetaboAnalyst 5.0.

## Figures and Tables

**Table 1:** Details of experimental cohorts.

Experiment	Group	Strain	Diet	Age	Time of Sac	Nutritional State
Age	Young	C57BL/6N	chow (Altromin 1314)	2 months	ZT6	4 h fasted
	Old	C57BL/6N	chow (Altromin 1314)	21 months	ZT6	4 h fasted
Diet	Low fat diet (LFD)	C57BL/6J	20 weeks 10% kcal fat (D12450Ji Research diets)	7 months	ZT6	4 h fasted
	High fat diet (HFD)	C57BL/6J	20 weeks 60% kcal fat (D12492i Research diets)	7 months	ZT6	4 h fasted
Lifestyle	Trained + chow	C57BL/6J	chow (4RF21 Mucedola, Settimo Milanese, Italy)	7 months	ZT11	End of the physiological fasting phase
	Sedentary + HFD	C57BL/6J	12 weeks 60% kcal fat (PF4051/D, Mucedola, Settimo Milanese, Italy)	7 months	ZT11	End of the physiological fasting phase

## Data Availability

Metabolomics data can be found in the supplementary material. Further information and requests for resources should be directed to and will be fulfilled by the corresponding author, Kenneth A. Dyar (kenneth.dyar@helmholtz-muenchen.de).

## Supplementary Materials

The following are available online at https://www.mdpi.com/article/10.3390/ijms22157958/s1.
